# Stereotactic Body Radiation Therapy Without Androgen Deprivation in a 92-Year-Old With Oligometastatic Stage IVA Prostate Cancer: A Case Report

**DOI:** 10.7759/cureus.93748

**Published:** 2025-10-02

**Authors:** Pericles J Ioannides, Shane A Tipton, Georg A Weidlich

**Affiliations:** 1 Radiation Oncology, Adventist Health System, Sonora, USA; 2 Medical Oncology, Adventist Health System, Sonora, USA; 3 Radiation Oncology, National Medical Physics and Dosimetry Company, Palo Alto, USA; 4 Physics, National Medical Physics and Dosimetry Company, Palo Alto, USA

**Keywords:** androgen deprovation therapy, elderly patient, nonagenarian, oligometastatic, pelvic lymph nodes, prostate cancer, quality of life, rectal spacer, sbrt, stereotactic body radiotherapy

## Abstract

We report a case of a 92-year-old elderly male with Stage IVA oligometastatic prostate adenocarcinoma to the pelvic nodes managed successfully with stereotactic body radiotherapy (SBRT) alone. The patient declined androgen deprivation therapy (ADT) with concerns about quality of life and reported side effects. He was treated with weekly SBRT targeting the prostate and pelvic lymph nodes without a rectal spacer, experiencing only Grade 1 urinary toxicity. The prostate-specific antigen (PSA) significantly declined from 12.05 to 0.16 ng/ml at the six-month follow-up. This case shows the feasibility and tolerability of SBRT as monotherapy in select elderly patients with regional risk node-positive prostate cancer.

## Introduction

The treatment of prostate cancer in nonagenarians is often complicated by frailty, comorbidities, and limited life expectancy, which constrains the use of systemic therapy. Current guidelines recommend ADT with or without radiation for node-positive disease. However, ADT can significantly impair the quality of life, exacerbate sarcopenia, and increase cardiac risk in elderly patients who may have low life expectancy due to advanced age and pre-existing comorbidities. The American Society of Clinical Oncology (ASCO) Guideline for Geriatric Oncology emphasizes tailoring cancer therapy to geriatric domains, such as frailty, functional status, and competing mortality risks, while minimizing toxicity and preserving independence [1]. Within this framework, SBRT without ADT emerges as a promising modality due to its convenience, reduced systemic burden, and favorable local control. This case demonstrates the role of once-weekly stereotactic body radiotherapy (SBRT) monotherapy as a potentially effective and tolerable treatment modality in carefully selected elderly patients who decline treatment with ADT.

## Case presentation

A 92-year-old male with Eastern Cooperative Oncology Group (ECOG) performance status of 1 was diagnosed with Stage IVA (cT1cN1M0) high-grade prostate adenocarcinoma. His comorbidities included a history of Atrial Fibrillation, type II diabetes, hypertension, and hyperlipidemia. He presented with an elevated prostate-specific antigen (PSA) of 7.42 ng/ml on October 5, 2023. Transrectal ultrasound (TRUS)-guided biopsy on February 5, 2024, revealed Grade Group 5 disease in all cores sampled. Prostate-specific membrane antigen positron emission tomography-computed tomography (PSMA PET-CT) imaging on March 7, 2024, for staging showed avidity in the right side of the prostate and two right pelvic lymph nodes. This pattern of disease was consistent with an oligometastatic presentation, defined as limited nodal involvement (≤5 lesions confined to the pelvis) without evidence of visceral or distant bony metastases, thereby distinguishing it from polymetastatic disease.

On June 3, 2024, the patient's PSA level increased to 12.05 ng/mL, at which point he was referred to medical oncology. The medical oncologist recommended ADT monotherapy; however, ADT was declined due to concerns about its side effects and impact on his quality of life. ADT is associated with substantial side effects in elderly patients, including fatigue, hot flashes, metabolic syndrome, bone and muscle loss, cognitive decline, and an increased risk of cardiovascular morbidity, which contributed to the patient's preference for SBRT monotherapy to avoid ADT.

PSMA PET-CT was repeated for monitoring on July 31, 2024, which confirmed persistent local disease in the prostate and regional nodes in the right pelvis. He was referred to radiation oncology to discuss the role of radiation therapy, where, due to concerns about daily travel, he elected treatment with SBRT over five sessions given once weekly. His baseline American Urological Association (AUA) score was 17 (moderate symptom score), urinary quality of life (QOL) score of 0 (Delighted), and sexual health inventory for men (SHIM) of 5 (severe erectile dysfunction). The elevated PSA value from June justified proceeding with treatment. A rectal spacer was discussed, but the patient declined due to concerns about undergoing any invasive procedures. 

A pre-treatment MRI was not obtained because the patient was unable to tolerate the time required for image acquisition. No fiducial markers were placed due to the patient's preference not to undergo another urologic procedure. CT simulation was performed with a pre-treatment fleet enema and a full bladder, as per the SBRT protocol. A Foley catheter was not required to delineate the urethra, as our standard clinical institutional protocol addressed dose homogeneity to ensure that the urethra remained under the dose tolerance, with a maximum dose of less than 42 Gy. The patient was evaluated and found to have severe erectile dysfunction; therefore, the neurovascular bundle was of no concern during treatment planning.

**Figure 1:**
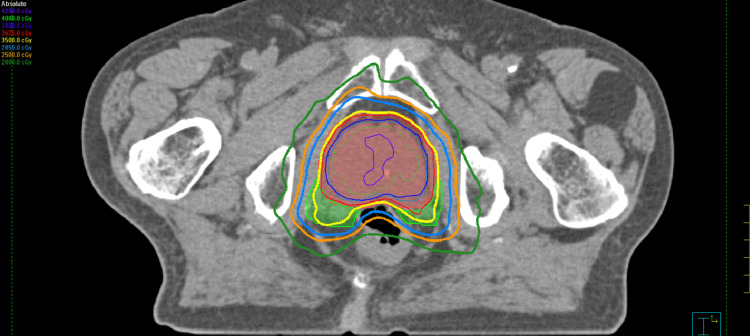
The dosimetric treatment plan with prescription dose treating the prostate gland and proximal seminal vesicles (35 Gy-yellow isodose line)

**Figure 2:**
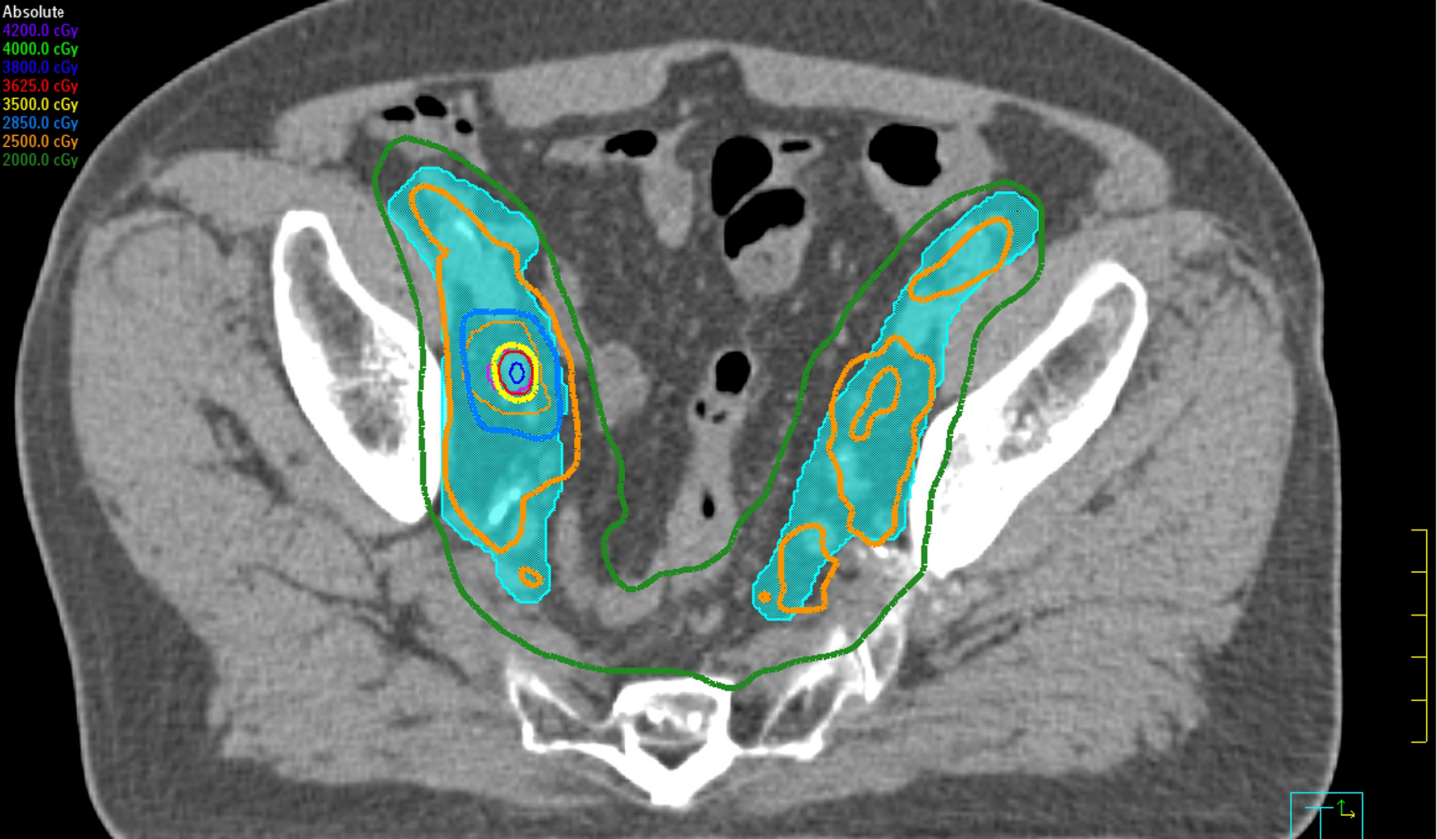
The dosimetric treatment plan with color-coded isodose lines (35 Gy-yellow isodose line)

**Figure 3:**
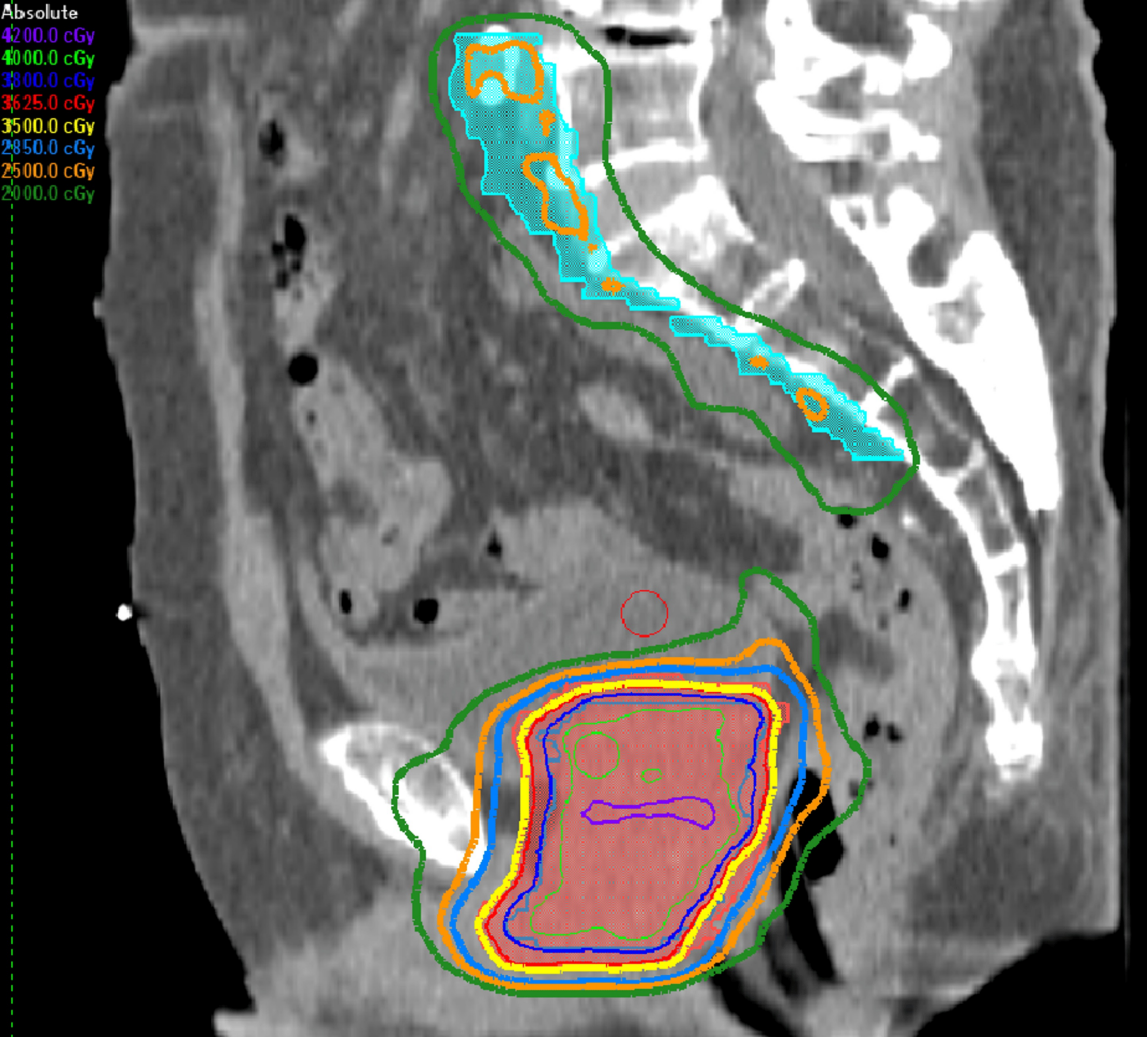
The dosimetric treatment plan with color-coded isodose lines (35 Gy-yellow isodose line)

He was treated with SBRT to a dose of 25-40 Gy in five fractions from August 26 to September 25, 2024, on an Elekta Infinity Linear Accelerator, including the Agility Head. Figures 1, 2, 3 illustrate the dosimetric treatment plan with color-coded isodose lines. He received 25 Gy in five fractions to the elective pelvic nodal volumes, including the internal and external iliac, presacral, and obturator lymph nodes, with simultaneous integrated boost to 35 Gy in five fractions to the involved right internal iliac nodes and at risk proximal seminal vesicles, followed by a 40 Gy in five fractions to the prostate in once weekly fractions without rectal spacer.

**Figure 4:**
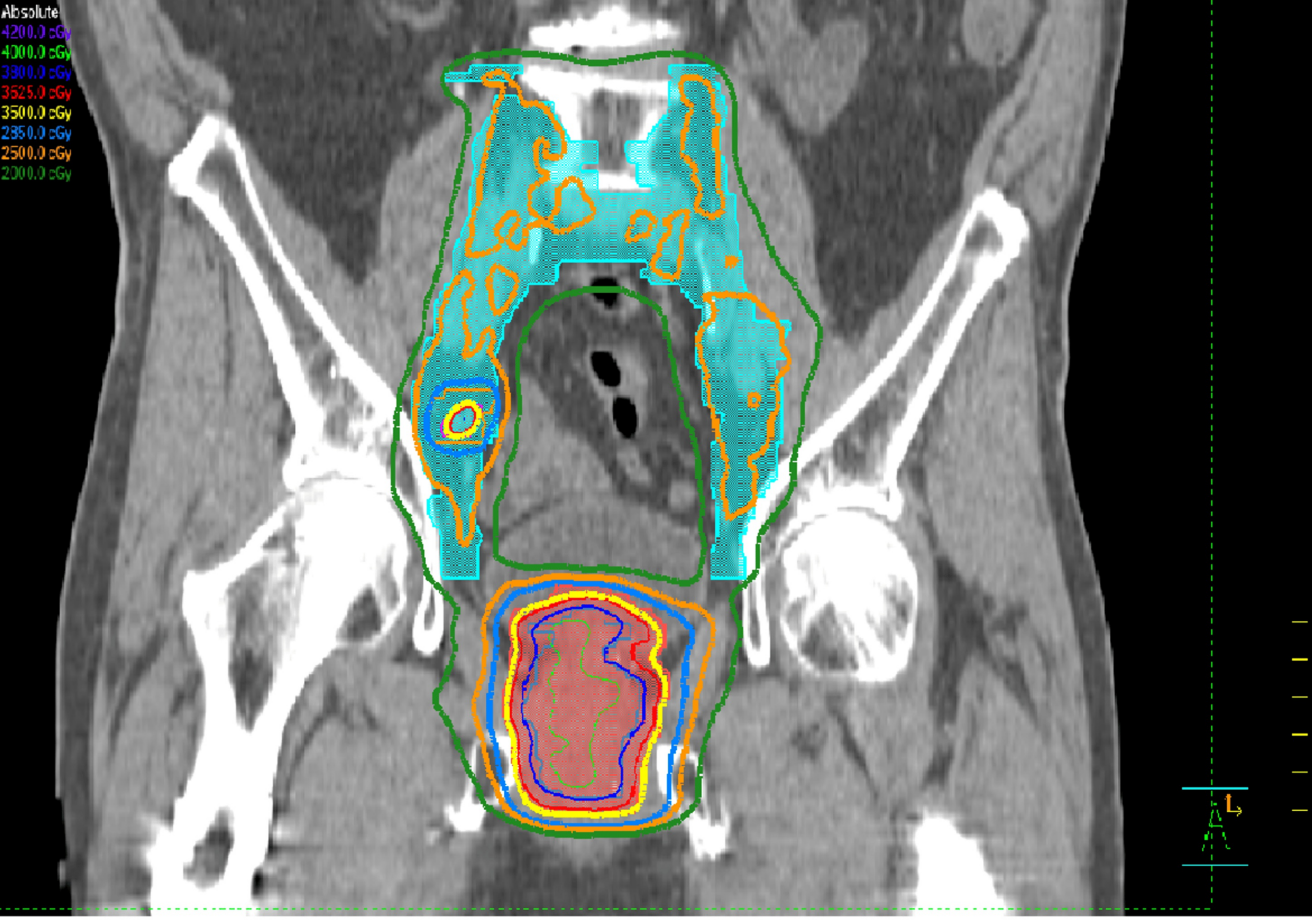
The approved 3D dose distribution (35 Gy-yellow isodose line)

**Figure 5:**
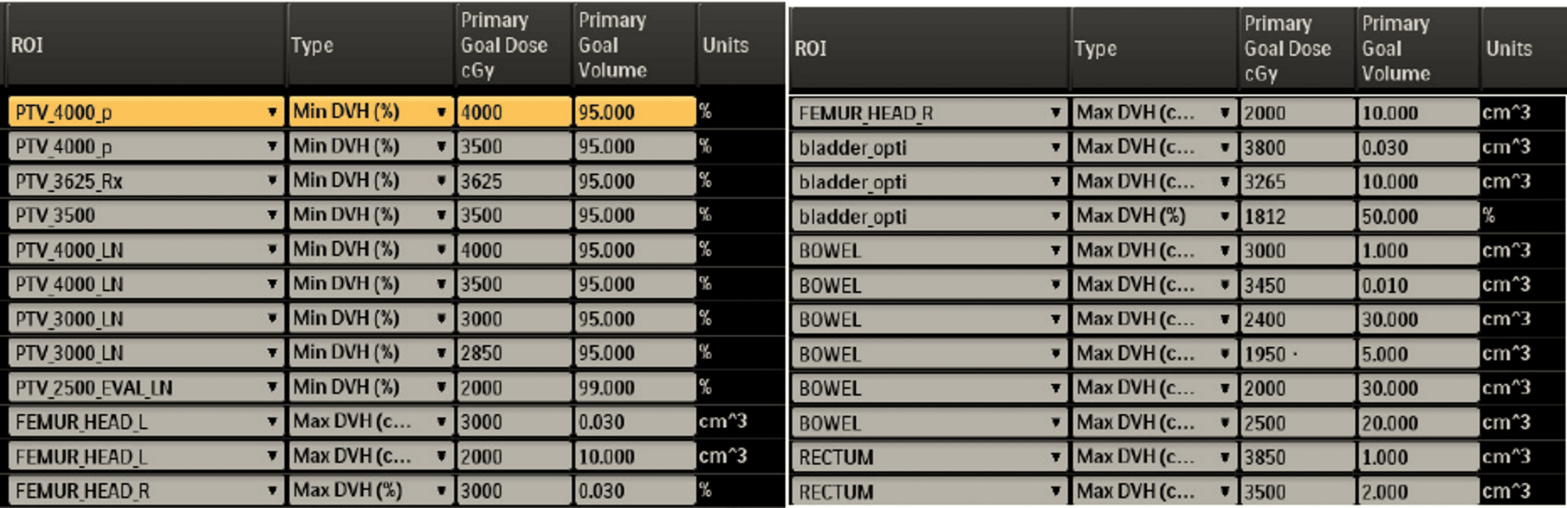
Planning objectives and organs at risk (OAR)

**Figure 6:**
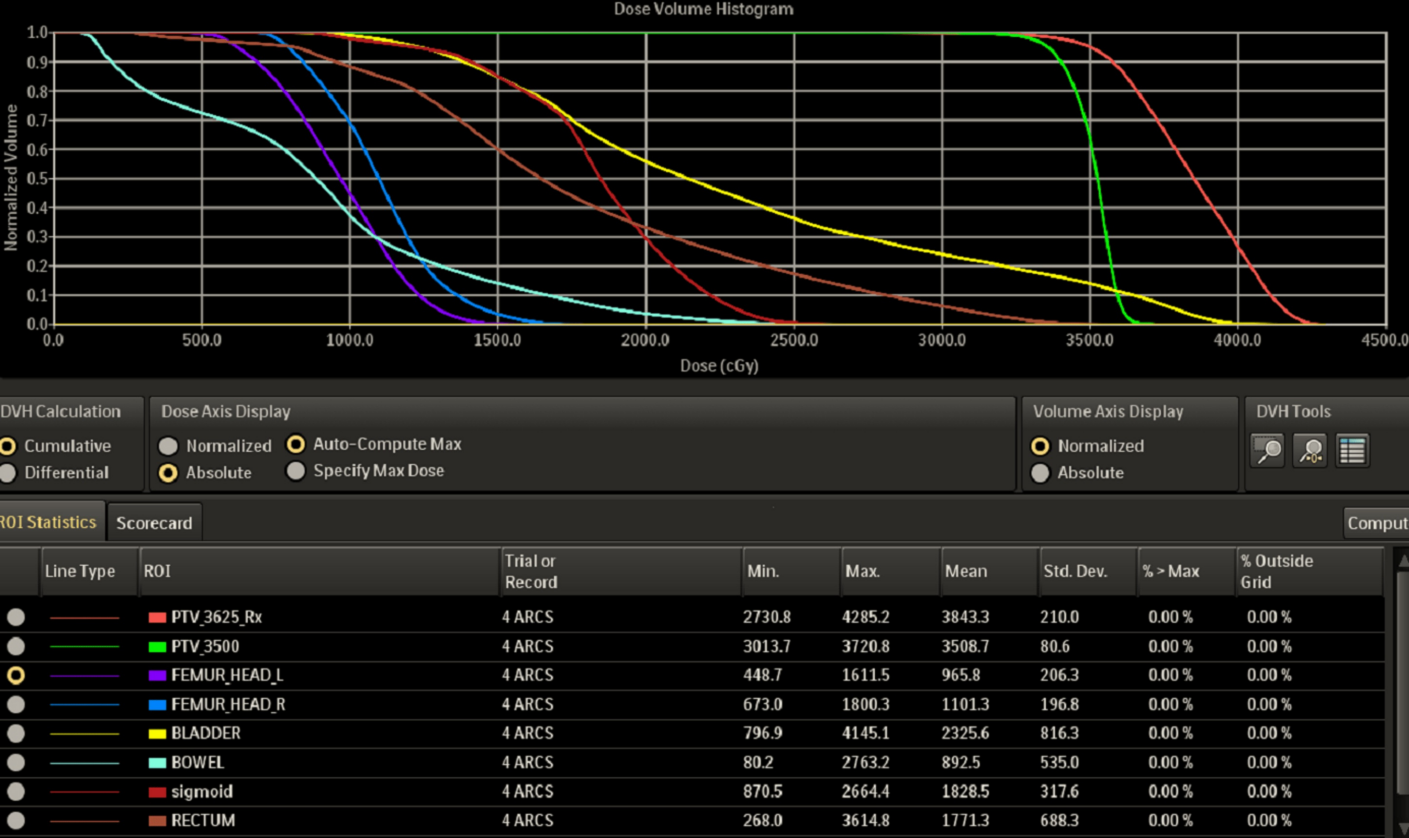
Dose Volume Histogram (DVH) including critical structure dose for the femoral heads, bladder, bowel, sigmoid, and rectum

**Figure 7:**
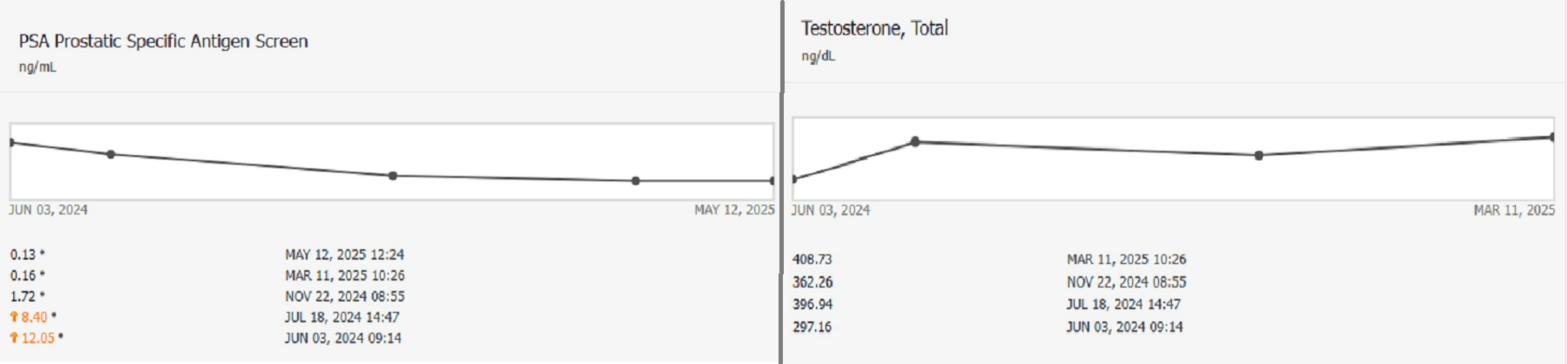
Prostate-specific antigen (PSA) and testosterone levels

The approved 3D dose distribution is shown in Figure 4, along with the Planning dose objectives and Dose Volume Histogram (DVH) in Figures 5, 6, respectively. The post-treatment PSA declined to 0.16 ng/mL six months post-treatment on March 11, 2025, at the six-month follow-up. The time-dependent PSA and corresponding testosterone levels are shown in Figure 7. 

During this short six-month follow-up, he experienced only common terminology criteria for adverse events (CTCAE) v 5.0 G1 dysuria and urgency, with no change in gastrointestinal or sexual side effects from baseline. EPIC-26 confirmed the preservation of urinary, bowel, sexual, and hormonal function with no significant change in AUA, QOL, or SHIM scores from baseline.

He now remains on surveillance PSA and testosterone monitoring every six months as per recommended guidelines for survivorship to establish long-term durability of results. 

## Discussion

This case report demonstrates the successful management of high-grade, node-positive regional prostate cancer with SBRT monotherapy in a nonagenarian who declined ADT. SBRT was tolerated well and resulted in a rapid decline in PSA, leading to early biochemical control of the disease. Most studies reported in the literature for the treatment of regional and distant metastatic prostate cancer, including systemic therapy in advancing or metastatic prostate cancer: evaluation of drug efficacy (STAMPEDE) [2], observation vs. stereotactic ablative radiation for oligomets trial (ORIOLE) [3], and stereotactic ablative radiotherapy for the comprehensive treatment of oligometastases trial (SABR-COMET) [4], advocate for systemic therapy combined with local treatment. Our patient demonstrates real-world evidence that SBRT alone may be sufficient in selected elderly and frail patients while preserving hormonal function.

The weekly SBRT regimen, supported by the prostate advances in comparative evidence-B trial (PACE-B) trial [5], allowed for a convenient and reduced treatment burden for our patients, who would otherwise not have been able to undergo treatment. The dose regimen of 25 Gy to the pelvic nodes, 35 Gy to the involved nodes, and 40 Gy to the prostate was selected based on evidence supporting the safety of using 25 Gy in five fractions for elective nodal irradiation elective nodal irradiation (ENI) [6]. This case adds to the novelty of demonstrating acceptable rectal dosimetry without a rectal spacer, aligning it with the growing literature on the safety of modern planning techniques that utilize an intrarectal catheter to minimize gaseous distension, and daily CBCT imaging to position the prostate interface and anterior rectal wall [7].

Additionally, this case report contributes to the feasibility of personalizing treatment planning, guided by informed discussion of patient preferences and comorbidities. Trials are increasingly emphasizing the role of imaging with PSMA PET-CT in staging and treatment planning for oligometastatic disease, allowing for improved localization and dose escalation to involved sites, and may impact biochemical recurrence [8,9]. Furthermore, SBRT is non-inferior to conventional fractionation in terms of failure-free survival, and late toxicity is similar in both treatment groups [10]. There is also data suggesting that the omission of ADT in patients with definitive therapy for unfavorable intermediate-risk patients may not compromise outcomes when local control is optimized [11]. This report contributes to the growing literature advocating for tailored, effective, and safe treatment strategies with less toxicity in the aging population of patients with prostate cancer [12].

## Conclusions

SBRT monotherapy may represent a convenient and tolerable treatment option for carefully selected elderly patients with high-grade, node-positive prostate adenocarcinoma who decline androgen deprivation therapy. In this case, a weekly SBRT regimen without a rectal spacer was well tolerated and demonstrated a significant early biochemical response. While this short interval is insufficient to infer durable disease control, and the risk of regional or distant relapse remains high in this setting, the initial favorable response highlights the potential role of SBRT monotherapy as a successful de-intensified, patient-centered strategy. Longer follow-up and additional validation with prospective data are needed to clarify its long-term efficacy and safety. 
